# Tensile Fracture Behaviour of Prismatic Notched Specimens of Cold Drawn Pearlitic Steel: A Macro- and Micro-Approach

**DOI:** 10.3390/ma18081690

**Published:** 2025-04-08

**Authors:** Jesús Toribio, Francisco-Javier Ayaso, Rocío Rodríguez

**Affiliations:** Fracture and Structural Integrity Research Group (FSIRG), University of Salamanca (USAL), E.P.S., Campus Viriato, Avda. Requejo 33, 49022 Zamora, Spain; fja@usal.es (F.-J.A.); rociorg@usal.es (R.R.)

**Keywords:** high-strength pearlitic steel, cold drawing, pearlite colonies, ferrite/cementite lamellae, stress triaxiality, constraint

## Abstract

This paper focuses on the study of the tensile fracture behaviour of prismatic notched specimens of cold drawn pearlitic steel, providing a *macro*- and *micro*-approach. Two types of notched samples with very different notch radius (*sharp* and *blunt* notches, PAA and PCC) and the same notch depth were studied, thereby allowing a study of the fracture behaviour under different levels of *stress triaxiality* (constraint) in the experimental specimen. The studied samples are machined from pearlitic steel wires taken from a real cold drawing chain, analysing the entire drawing process, from the initial base material (*hot rolled bar*; not cold drawn at all) to the final commercial product (*prestressing steel wires*; heavily cold drawn), including two intermediate stages in the manufacture chain. The aforesaid specimens were subjected to tensile fracture tests and analysed at macroscopic and microscopical level using the scanning electron microscope (SEM), thereby obtaining micrographs of the different areas appearing in the specimens under study and assembling full micro-fracture maps (MFMs) of the fractured area. The aim of the research is to analyse the macro- and microscopic changes produced by the variation in stress triaxiality state (constraint), along with the different fracture processes. The first relevant finding is the increase in fracture path deflection for higher drawing degrees, and for greater triaxiality levels associated with sharp notches. Another finding is the variation in area of the different fracture zones, i.e., outer crown (OC), fracture process zone (FPZ) and intermediate zone (Z_INT_), which are characterised by their specific micro-mechanisms, micro-void coalescence (MVC), cleavage (C) and special (*large*) micro-void coalescence (MVC*). The higher the stress triaxiality level, the larger the area occupied by the Z_INT_ in the fracture process. The fracture behaviour tends to unify along with the degree of drawing, with less dependence on the state of triaxiality imposed on heavily drawn wires. Results have been obtained in which the increase in triaxiality, imposed by the smaller radius of curvature of the notch (*sharp notch*), as well as the greater degree of drawing of the wires, cause the fracture process to place the FPZ at the notch tip. It is demonstrated that the variation in stress triaxiality and the drawing degree can generate different locations of the fracture initiation zone (FPZ).

## 1. Introduction

Wire drawing produces microstructural changes in pearlitic steel, such as a reorientation of the pearlite colonies and the sheets that make them up in the direction of the longitudinal axis of the wire, a progressive decrease in interlaminar spacing, and an increase in the slenderness of the colonies [1].

The cold drawing process generates an increase in yield strength and ultimate tensile strength (UTS) of steel through a strain hardening mechanism. Numerous scientific works have analysed the relationship between microstructural evolution in pearlitic steels during cold drawing and the strength and ductility obtained after the manufacturing process, e.g., those carried out by Embury and Fisher [2] and Langford [3] on the drawing and deformation of pearlite, the review of data on the role of interlamellar spacing by Ridley [4], and the study of the important role in fracture of the previous austenite grain by Lewandowski and Thompson [5].

It is worth mentioning key studies carried out during the 1990s by Nam and Bae [6], who studied the austenitic previous microstructure, Toribio and Ovejero [1] who established the microstructural evolution of pearlite colonies and lamellae during the drawing process., and Nam et al. [6] on the microstructural evolution of pearlite in cold drawing, along with papers on the key role of non-metallic inclusions [7]. All these microscopic features and peculiarities can affect the posterior fracture behaviour of progressively cold-drawn pearlitic steel wires.

With regards to fracture behaviour, previous studies [8] show that cold drawn pearlitic steel exhibits anisotropic fracture behaviour, showing, on the one hand, the role of microstructural delamination prior to the generation of the fracture process zone (FPZ) and, on the other hand, the influence of both the microstructural changes and the micro defects generated by the drawing process. In the matter of subcritical fatigue, anisotropy also appears in the form of *locally anisotropic fatigue behaviour*, although a globally isotropic fatigue behaviour does exist [9]. In this way, a *local mixed-mode* appears during fatigue cracking, in the same manner as occurs when an external mixed-mode is applied [10,11,12].

Currently, there are numerous investigations that focus on the study of fracture in tests with notched specimens [13,14], some with specimens similar to those in the present study, i.e., prismatic notched, in different materials [15,16,17], and more specifically with the same types of specimen in different steels [18,19].

This article focuses on the mechanical study and fracture of rectangular (prismatic) section notched samples. The specimens have two types of notch with very different radii (*sharp* and *blunt* notch) and the same notch depth; this allows study of fracture behaviour under different degrees of stress triaxiality (constraint). These specimens have been obtained by machining from cylindrical samples corresponding to a real wire drawing line in industry. The study focuses on the initial undrawn wire rod (no previous plastic deformation), two intermediate steps (medium degree of plastic deformation) and the final product or commercial prestressed steel (high degree of plastic deformation). The analysis is completed by an examination of the fracture behaviour of the different specimens via scanning electron microscopy (SEM) techniques, as well as by image analysis, using AnaliSIS, i.e., qualitative and quantitative fractography.

The influence of triaxiality on fracture processes has been the subject of numerous recent investigations on many different types of materials, including steels and rocks [20,21]. In this study, the aim is to clarify the influence of triaxiality, its dependence or not on microstructure, as well as on the variation in fracture path and/or the modification of different zones of the fractographic surface. The importance of improving the understanding of the role of triaxiality in fracture processes lies in the fact that it will improve the prevention and prediction of the failure of materials and may even be used in the modification of mechanisms that prevail in the fracture, or in the formation of its microstructure.

## 2. Experimental Procedure

Eutectoid steels have a completely pearlitic structure. The eutectoid iron–iron carbide reaction to form pearlite is called a pearlitic or eutectoid reaction, as described in Expression (1).

(0.77 wt% C) → (0.022 wt% C) + Fe3C (6.70 wt% C)(1)

**Figure 1:**
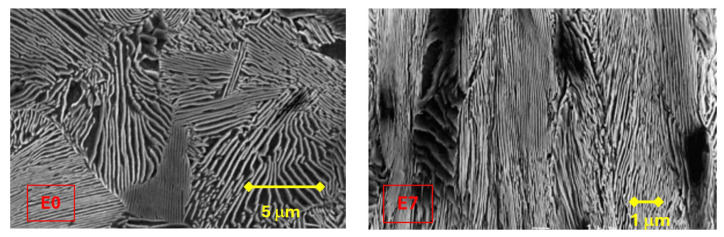
Pearlitic steel: initial wire rod and last pass of the drawing process.

The pearlitic microstructure is thus composed of alternating ferrite and cementite lamellae, which are grouped in colonies depending on their direction (Figure 1).

In the following study, eutectoid pearlitic steel, belonging to a real drawing chain formed by seven drawing rows, was used to analyze the initial wire rod and the commercially prestressed steel wire. The nomenclature used to identify the analysed wires consists of letters that identify the steel casting to which each belongs (in this case, the E family). The chemical composition of the type E steel used is detailed in Table 1. The number in the nomenclature of the steel will represent the step of the drawing process to which it belongs, number 0 being for the initial wire rod, 7 for the final product, and numbers 3 and 4 to identify the wires corresponding to the two intermediate steps. The mechanical properties of each of the wires studied are indicated in Table 2.

**Figure 2:**
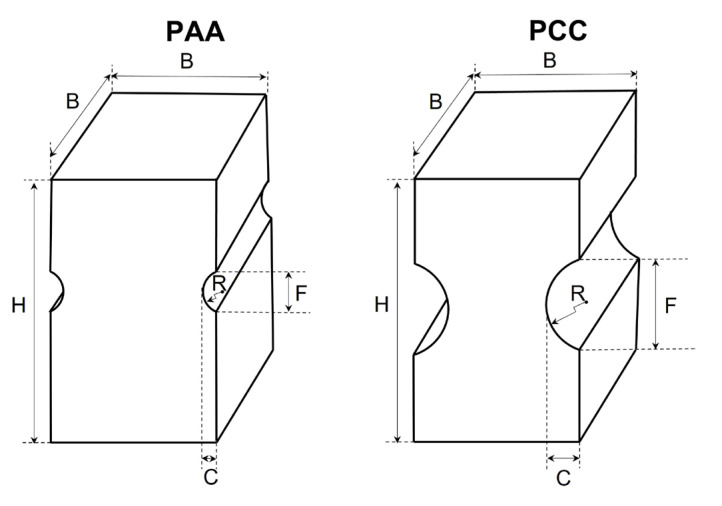
Scheme of the specimens with PAA and PCC notch geometries.

The drawing process produces a decrease in section and an increase in length, generating microstructural changes that lead to a change in the mechanical properties of the material, increasing the yield strength, the cumulative plastic strain and the UTS. In the present study, prismatic notched specimens (PAAs and PCCs) with the geometries shown in Figure 1 are used. The relationships for machining have been established in relation to the diameter (φ) of each of the steps in the drawing process to which the wire belongs (Figure 2). The measurements on which this relationship is established are the depth of the notch (C), the radius of the notch (R, i.e., the radius of curvature of the circumference that defines the profile of the notch at the tip of it) and the thickness of the prismatic specimen (B) (Table 3). The dimensions chosen for the specimens are based on previous research [22].

**Table 1:** Chemical composition (wt%) of steels in family E (the balance is Fe) [23].

C	Mn	Cr	Si	V	P	S	Al
0.79	0.68	0.22	0.21	0.06	0.01	0.01	0.003

For the microstructures to be visualized in the scanning electron microscope (SEM), the samples were prepared by embedding them in phenolic resins, polishing, and grinding until a specular surface was obtained, which was then treated with a 3% solution of Nital.

**Table 2:** Dimensions and mechanical properties of the steels analyzed [23].

Steel	*D* (mm)	*E* (GPa)	*σ*_Y_ (GPa)	*ε* ^P^_cum_	*σ*_R_ (GPa)
E0	11.03	199	0.72	0.00	1.23
E3	8.21	192	0.93	0.42	1.41
E4	7.49	196	1.02	0.78	1.50
E7	5.04	208	1.49	1.57	1.83

The SEM used is a JEOL model JSM-5610 LV (JEOL, Peabody, MA, USA), capable of generating secondary electron and accelerated backscattered electron images. The Soft Imaging System GmbH (Muenster, Germany), consisting of a computer and the image analysis software AnalySIS, was used in order to measure the different zones in the fractographies obtained.

**Table 3:** Geometry of the specimens studied [23].

Specimen	R/ϕ	C/ϕ	B/ϕ
PAA	0.022	0.060	0.617
PCC	0.220	0.057	0.617

## 3. Experimental Results

**Figure 3:**
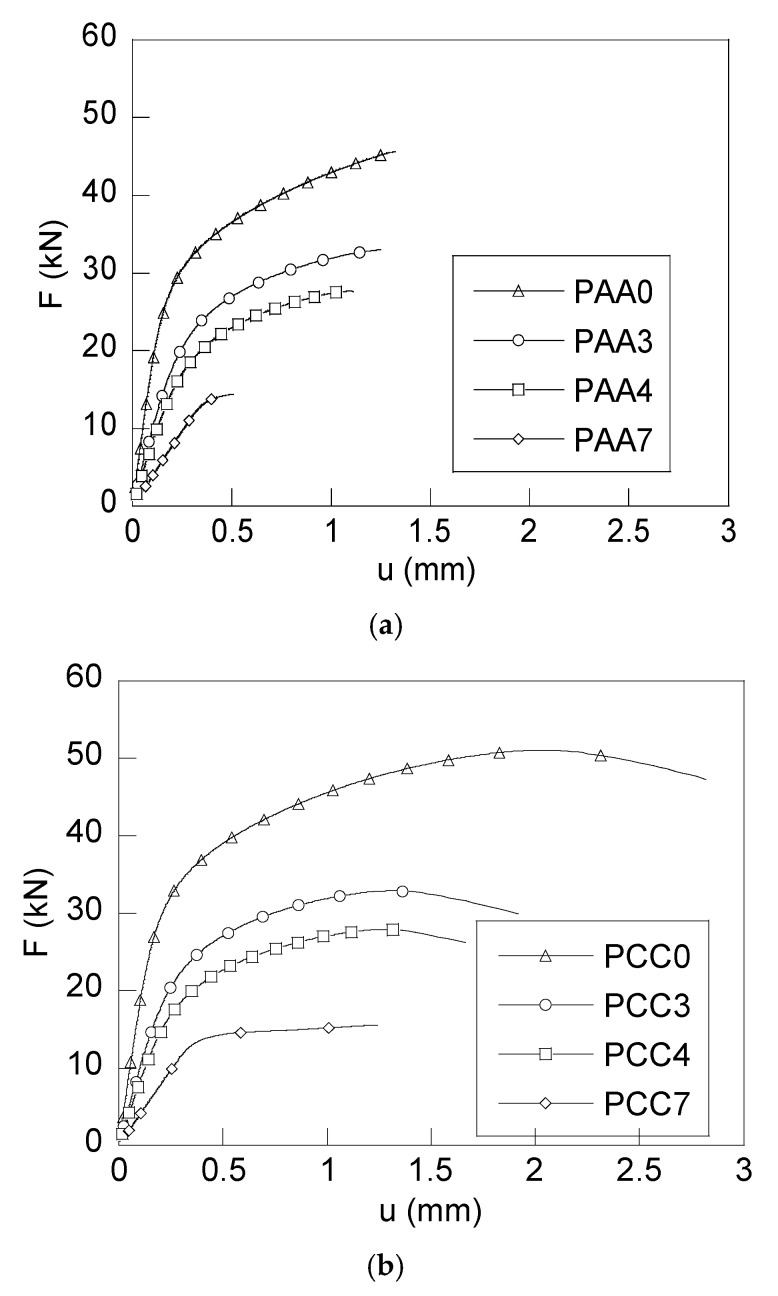
F–u curves of tensile tests, (**a**) PAA-type specimens, (**b**) PCC-type specimens.

From the tests carried out for each type of notch and each step of the drawing process, the load–displacement curves (*F–u*) were obtained and represented in Figure 3. The tensile fracture tests were carried out by imposing a crosshead speed of 0.01 mm/s in the PAA-type specimens and of 0.025 mm/s in the PCC-type specimens.

**Figure 4:**
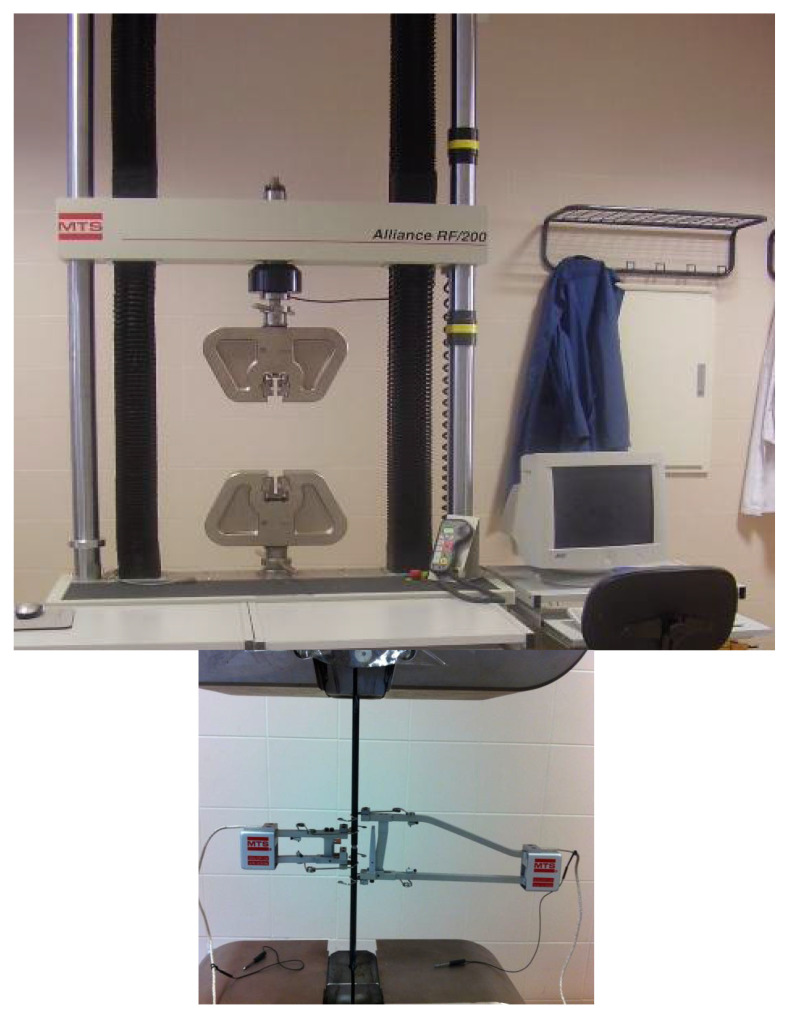
Servomechanical testing machines MTS RF/200 (MTS Systems Corporation, Eden Prairie, MN, USA).

The specimens obtained from the wires, belonging to different steps in the cold drawing process, have been produced using a precision grinding process. This mechanical process ensures that the specimen surfaces have average roughness values (Ra) of less than 0.25 μm. The notched specimens have been tested under tensile stress, until breakage, in the servomechanical testing machines MTS RT/100 and MTS RF/200 (Figure 4). For this purpose, the specimens, approximately 30 cm long, were placed between the grips of the machine, which are perfectly aligned with each other, which means that the specimen also *remains aligned during the test* and, therefore, the described specimens are *not* affected by possible bending stresses. Two axial dynamic strain gauges with measuring bases of 25 and 50 mm were placed on each specimen, and more specifically in the vicinity of the notch. Three specimens were tested for each wire pitch analyzed and for each type of notch, the results obtained being the average.

From the analysis of these *F*–*u* curves, the corresponding stiffness k for each particular specimen was evaluated, obtaining a similar stiffness in the specimens belonging to the same drawing step and a reduction in this parameter as the degree of steel cold drawing increases. In the analysis of the load corresponding to the elastic limit of the specimens (*F*_Y_), behaviour analogous to the stiffness has been observed, i.e., similar in specimens obtained from the same drawing step and decreasing in the successive steps.

Table 4 gives the load corresponding to the elastic limit of the samples (*F*_Y_), together with the stiffness of the specimens (k). As seen in Figure 3 and Table 4, the higher the cold drawing degree in each steel wire, the lower the elastic limit and the stiffness during the tensile fracture tests, a behaviour that is associated with the decrease in the wire diameter after cold drawing.

Steel, like other materials, can exhibit brittle or ductile fracture behaviour. The first type (brittle) is characterized—fractographically—by a micro-mechanism that presents a topography called cleavage (C), and the second type (ductile) shows a fracture micro-mechanism known as micro-void coalescence (MVC).

**Figure 5:**
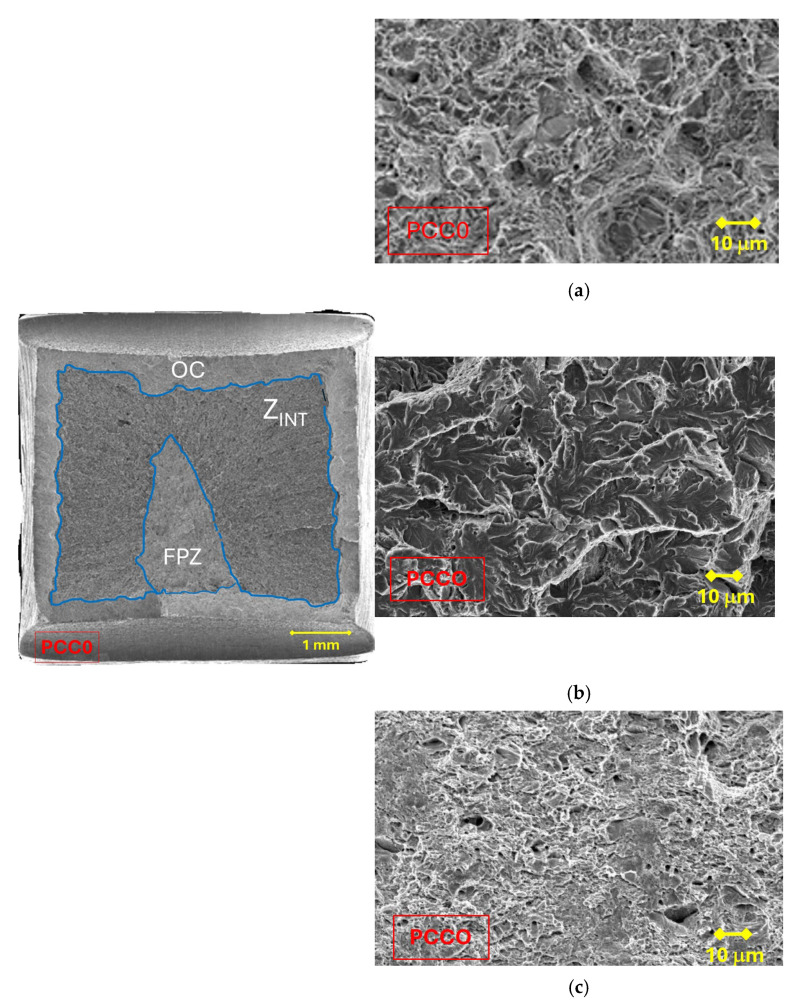
Fracture surface of the specimens; (**a**) outer crown (OC), (**b**) intermediate zone (Z_INT_) and (**c**) fracture process zone (FPZ).

In order to carry out the fractographic analysis, three tensile tests were performed for each case study, from the selected drawing process and type of notch. The tested samples were taken to the final fracture by total separation of surfaces and, subsequently, their fracture surfaces were examined by scanning electron microscopy (SEM). In the analysis of the different fracture surfaces, three zones can be observed (Figure 5), in which different fracture micro-mechanisms are presented: the fracture process zone (FPZ), intermediate zone (Z_INT_) and outer crown (OC).

**Figure 6:**
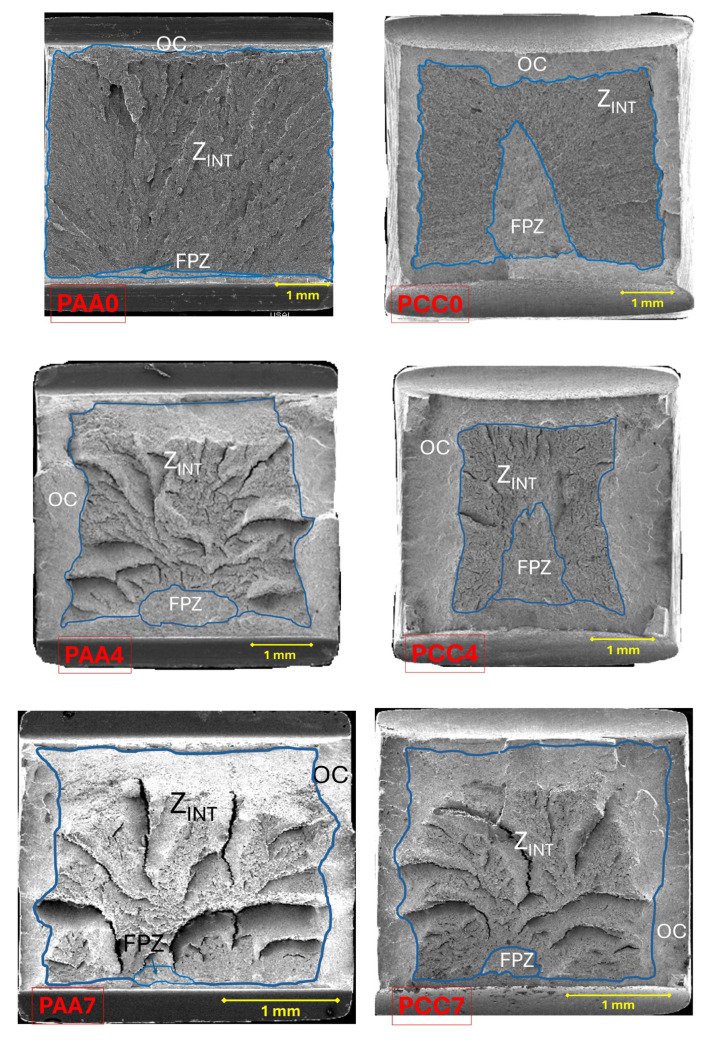
Fractographic surfaces of the different types of PAA and PCC notches.

The studied specimens behave in the same way, with the type of notch being responsible for one or another behaviour during fracture. Figure 6 shows the fracture surfaces obtained after carrying out the tensile fracture tests with the different specimens. Three different steel wires (E0, E4 and E7) are shown in the figure, and both notched geometries (sharp notch PAA and blunt notch PCC) are considered, so that the two main variables are analyzed: (i) the cumulative plastic strain or cold drawing degree in the materials; (ii) the stress triaxiality (constraint) through the notch geometry.

**Figure 7:**
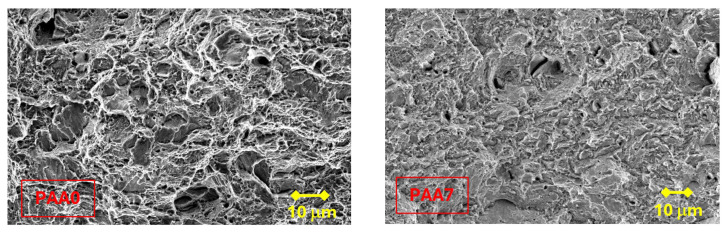
Fracture process zone: MVC*.

In all the specimens analyzed in the present paper, the following zones can be differentiated: a FPZ constituted by *large* micro-void coalescence (MVC*); the Z_INT_ formed by cleavage (C) that decreases with the degree of drawing, in favor of the increase in MVC; finally, the OC made up of MVC. In the FPZ, a decrease in the size of the micro-voids was observed as the degree of drawing increases, as shown in Figure 7.

**Figure 8:**
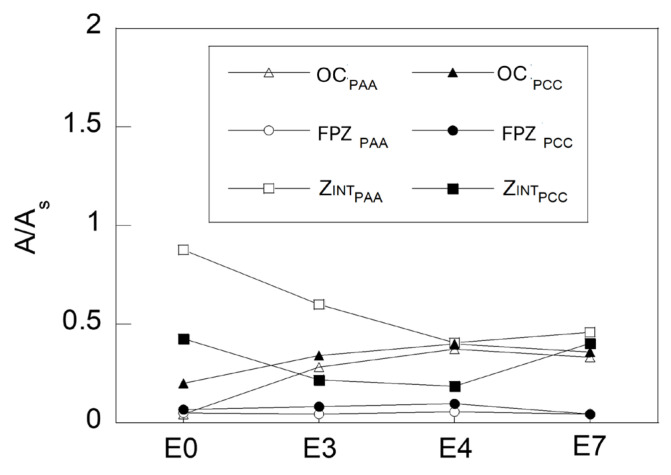
*A*/*As* of the different specimens.

Figure 8 comparatively represents the values corresponding to the different characteristic zones of the fracture surfaces obtained from the tensile fracture tests on the notched samples. The areas of the different zones of the fracture surface have been measured and are represented in a dimensionless form in Figure 8, taking into account the ratio A/A_S_, where A the area of each zone measured (OC, Z_INT_ and FPZ) and A_S_ the area of the net section of each specimen considered, i.e., the section of the specimen measured at the bottom of the notch or minimum cross section.

In both types of notch, analogous behaviour is observed in terms of the increasing trend in the OC area and the decreasing trend in the Z_INT_ as the drawing process progresses. The exception in both types of notch is the commercial prestressing steel wire, the last step in the drawing process, which shows a trend contrary to that mentioned, possibly due to the thermomechanical stress relaxation treatment to which this material has been subjected once drawn. In both types of notch, the small variation in the FPZ area should also be noted.

The PAA-type specimens show a fracture mechanism in which fracture begins (FPZ) in one of the notches and progresses towards the interior of the specimen until it reaches the opposite notch, as shown by the cleavages located in the intermediate zone (Z_INT_); this is caused by small irregularities in the machining of the two notches for the same test piece (one and its opposite). These slight deviations in the “real” machining cause stress and strain concentrations with slight differences on both sides of each of the PAA-type samples, which affect the real location of the FPZ. If the notches present exactly identical geometries, it is possible to expect a simultaneous fracture initiation in both notches, progressing towards the center of the corresponding sample.

**Figure 9:**
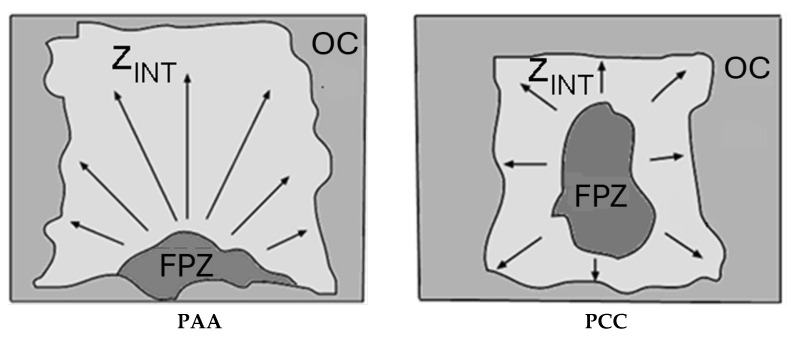
Diagram of the fracture mechanisms associated with each type of notch, in which arrows indicate the directions of cleavage propagation (i.e., the unstable brittle fracture stage).

The fracture mechanism observed in the PCC-type specimens is different from the previous examples, since the beginning of the fracture is located in the central zone of the wire and subsequently advances towards the outside of the specimen, as shown in Figure 9. However, in the specimen PCC7 machined from the prestressed steel wire, the behaviour of the specimen is similar to that of the PAA type specimens, i.e., the FPZ is located close to one of the notches. This may be due to the machining itself, as well as the special microstructural arrangement of the pearlitic colonies in the prestressing steel wire and its influence on the anisotropic behaviour in the fracture.

The cold drawn pearlitic steel under study in the present paper exhibits two types of fracture: (i) the *isotropic fracture*, in which the fracture surface is fully contained in a plane transverse to the longitudinal wire axis (or cold drawing direction), and (ii) the *anisotropic fracture,* in which the fracture surface presents irregularities in the form of local deflections in a direction perpendicular to the transverse plane, following a fracture path oriented quasi-parallel to the wire axis or cold drawing direction, i.e., the minimum resistance path (*weakest path*).

**Figure 10:**
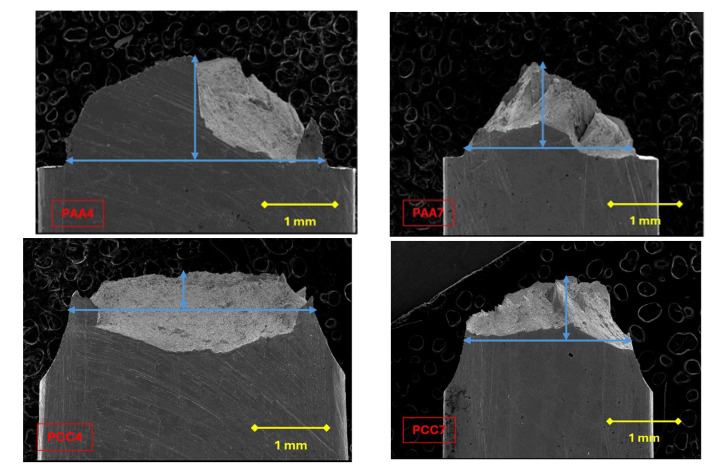
Anisotropic fracture behaviour of the PAA and PCC specimens; arrows indicate the extension (horizontal) and height (vertical) of the *anisotropic fracture region* associated with fracture path deflection and mixed mode propagation.

In the notched specimens under study, local anisotropic fractures occur in the last steps of the drawing process, as shown in Figure 10, this effect being observable in the fourth and last step of the drawing process in the PAA type specimens. However, in the PCC specimens, only the specimen machined from the PCC7 prestressed steel wire exhibits this anisotropic behaviour (in the fourth step of said process, a negligible *fracture path deflection* is produced).

**Figure 11:**
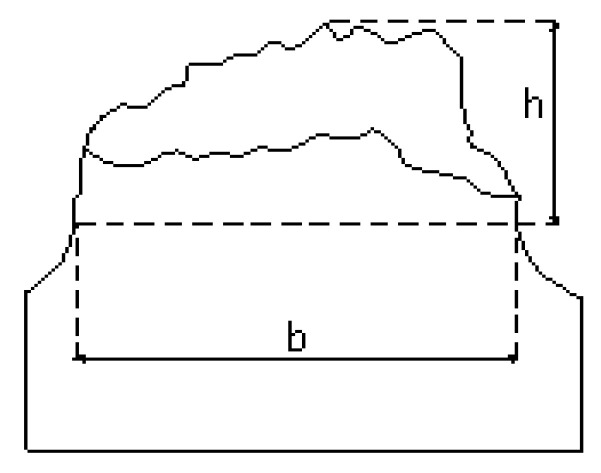
Fracture path deflection measurement scheme.

To quantitatively study the deflection of the fracture path, the distance b between notch tips is followed by the maximum height h reached by the anisotropic fracture, this being the vertical measured from the notch bottom up to the highest point of the anisotropic fracture surface (Figure 11); in this way, the dimensionless parameter h/b reflected in Table 5 is obtained, in which it is observed that type AA notches (small sharp notches) induce a greater anisotropic behaviour in fracture, reflected in the formation of large crests and valleys.

**Figure 12:**
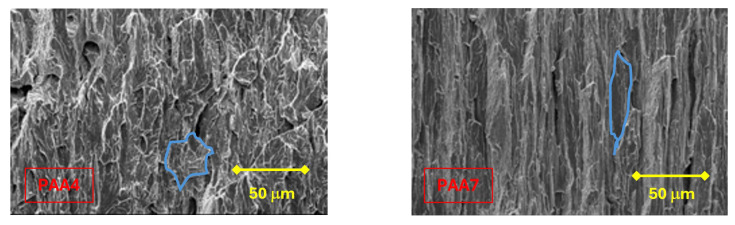
Cleavage facets (whose boundaries are drawn in blue lines) in the fracture surfaces of the vertical walls: specimens PAA4 and PAA7. The latter shows an *enlarged and oriented cleavage facet*, whose orientation and enlargement follows the wire axis or cold drawing direction (vertical side of the micrograph), a clear indication of the highly anisotropic nature of the most heavily drawn pearlitic steel (commercial prestressing steel wire).

In the vertical walls of the fracture surface of the specimens that show anisotropic behaviour, *enlarged and oriented cleavages* are observed quasi-parallel to the drawing direction. Such an orientation is more pronounced in the test pieces of the wires belonging to the last step in the drawing process (Figure 12).

## 4. Discussion

In the PCC samples, a clear elongation before fracture is observed, higher than that appearing in the case of the PAA. This is caused by the notch radius: the larger the notch radius, the greater the degree of plastic deformation before failure that the particular specimen is capable of withstanding. The same is reflected in the fractographic study: the samples with blunt notches (PCC) show a greater outer crown surface (OC) or ductile shear lip. On the other hand, the sharp notch (PAA) specimens show a greater amount of fracture surface due to cleavage.

Once again, the notch tip radius is responsible for the geometric location of the fracture process zone (FPZ). The PAA-type samples (*sharp notches* similar to a crack) produce a large concentration of stresses at the notch tip and its vicinity, inducing the fracture to incubate and propagate from such a place. On the other hand, the PCC-type samples (*blunt notches*) behave similarly to a smooth sample under necking during a standard tension test, i.e., the concentration of stresses occur in the center of the sample, where the FPZ is formed and fracture initiates.

The fracture site with variations in the state of stress triaxiality has been and is the subject of study in numerous investigations [24]. The importance of predicting the fracture initiation situation in a material can not only lead to a great improvement in failure prevention methods but can also serve to control fracture processes.

Numerous investigations have focused on the study of stress triaxiality to achieve the objective of predicting and controlling failure in different types of materials [20,21], to understand fracture processes in rocks [25], and even to control the final microstructure of materials during their formation process [26,27].

A decrease in the size of the micro-voids in the FPZ has been observed as the degree of cold drawing in each steel wire increases, as well as an increase in the slenderness of the cleavages (associated with anisotropic brittle fracture) in the vertical walls of heavily drawn steels, as the drawing process increases.

The observations above are associated with the microstructural changes suffered by the steel in the cold drawing process, such as the decrease in interlaminar spacing, the orientation of the pearlite (ferrite/cementite) sheets, and the increase in slenderness of the pearlite colonies [1].

Stress triaxiality variation also plays a large role in recent research on hydrogen propagation in fracture processes. Recent research has shown that increasing triaxiality promotes hydrogen concentration in the notch [28], or that the failure criterion is independent of triaxiality in the case of hydrogen-induced failure [29]. The object of the present research provides knowledge of fracture progression in non-aggressive environments that can serve as a basis comparative or complementary to these macro- and micro-fractographic studies.

Recent studies have shown that increasing triaxial stress results in a decrease in the critical load in steel due to pronounced interface decohesion at the microstructural level [30]. The great importance of stress triaxiality means that the study of stress triaxiality variation is used as a guide to propose a new ductile fracture model based on the decomposition of the stress vector [31].

The increase in triaxiality can generate a higher rate of voids at the microstructural level [30]. In the material under study, pearlitic steel, the creation of voids would occur at the boundaries of pearlite colonies, as this is where fracture propagation takes place, as areas of greatest microstructural weakness [32,33]. Increasing the rate of voids between perlite colonies would lead to easier and faster separation of the colonies.

This fact is demonstrated by the increase in the Z_INT_ specimens of higher triaxiality, PAA, which is composed of micro-mechanisms typical of brittle fractures, such as cleavages. The increase in the fracture surface of brittle-type micro-mechanisms is lower in strongly drawn steels in both types of specimens analysed.

Stress triaxiality is, therefore, not so easily capable of producing that separation, i.e., the generation of voids, which favours a more brittle type of fracture. This result indicates how microstructural reorganisation that occurs in the drawn pearlitic steel makes the fracture behaviour of the material less dependent on the state of stress triaxiality.

The notch tip radius, together with the degree of previous plastic deformation of the material used (obtained during drawing), influences the locally anisotropic fracture of the material. The anisotropic behavior in fracture becomes greater as the notch tip radius decreases and the yield stress of the material increases (higher degree of drawing). The increased deflection of the fracture path occurs in heavily drawn steels due to the microstructural reorganization of the lamellae forming the pearlite colonies, as well as the increased slenderness of the colonies.

Both changes in orientation and shape are in favor of the direction of the drawing process. In the specimens subjected to higher triaxial stress, i.e., PAA with smaller bending radius, there is a higher deflection of the fracture path of up to 34% more in strongly drawn steel than in PCC test specimens with larger bending radius.

In steels belonging to intermediate cold drawing degrees, the difference in fracture path deflection between specimens is even greater, reaching up to 64%. The smaller difference in fracture path deflection shown by the heavily cold drawn steel wires of the different specimens, PAA7 and PCC7, is due to the lower influence of the triaxiality stress since, in these deformation-hardened microstructures, it is more difficult to generate micro-voids. In addition, the voids are generated, as mentioned above, at the boundaries of colonies and will therefore also show a preferential orientation of creation. Stress triaxiality, therefore, has a greater influence as an enhancer of fracture path deflection in slightly cold drawn steels.

## 5. Conclusions

The PAA and PCC type samples show similar variations in stiffness and load corresponding to the elastic limit, producing in both geometries a decrease in these parameters as the drawing degree increases. The exceptions to the trend are the PAA7 and PCC7 samples due to the thermo-mechanical treatment inherent to the manufacturing process of the material.

The PAA and PCC type specimens show similar trends in the area variation of the different zones present in the fracture surface, with the area of the outer crown (OC) zone increasing as the wire drawing level increases. The exception is the commercial prestressed steel wire, as a result of the thermo-mechanical stress relaxation treatment to which the steel has been subjected.

The fracture process zone (FPZ) differs in terms of its spatial location within the samples studied, being in the *periphery of the sample* (*notch tip vicinity*) for specimens with a small notch tip radius (*sharp notch specimens* PAA) and, on the other hand, FPZ is located in the *center of the sample* (*specimen axis*) for specimens with a large notch tip radius (*blunt notch specimens* PCC).

In the specimens subjected to higher triaxial stress., i.e., PAA with smaller bending radius, there is a greater deflection in the fracture path, up to 34% greater in the drawn steel than in the PCC specimens with larger bending radius. In steels belonging to intermediate cold drawing degrees, the difference in fracture path deflection between specimens is even greater, up to 64%.

The smaller difference in fracture path deflection shown by the heavily drawn wires of the different specimens, PAA7 and PCC7, is due to the lower influence of stress triaxiality, since, in these strain-hardened pearlitic microstructures, it is more difficult to generate micro voids that accelerate fracture.

The microstructural reorganisation taking place in the progressively cold drawn pearlitic steel wire makes the fracture behaviour of the material less dependent on the stress triaxiality state. Therefore, in this case microstructural evolution plays a more important role in fracture than the stress triaxiality itself.

## Figures and Tables

**Table 4:** Values of F_Y_ and k for the test specimens.

	PAA		PCC	
	F_Y_ (kN)	k (kN/mm)	F_Y_ (kN)	k (kN/mm)
E0	19.886	182.93	21.191	179.03
E3	13.975	96.370	13.546	96.952
E4	11.199	79.692	11.163	79.200
E7	13.253	37.786	13.229	38.173

**Table 5:** Measurements of the deflection of the fracture path.

	PAA4	PAA7	PCC4	PCC7
b/h	0.39	0.53	0.14	0.35

## Data Availability

The original contributions presented in this study are included in the article. Further inquiries can be directed to the corresponding author.

## References

[1] Toribio J., Ovejero E. (2000). Composite microstructure of cold-drawn pearlitic steel and its role in stress corrosion behavior. ASM J. Mater. Eng. Perform..

[2] Embury J.D., Fisher R.M. (1966). The structure and properties of drawn pearlite. Acta Metall..

[3] Langford G. (1977). Deformation of pearlite. Metall. Trans. A.

[4] Ridley N. (1984). A review of the data on the interlamellar spacing of pearlite. Metall. Trans. A.

[5] Lewandowski J.J., Thompson A.W. (1986). Effects of the prior austenite grain size on the ductility of fully pearlitic eutectoid steel. Metall. Trans. A.

[6] Nam W.J., Bae C.M. (1995). Void initiation and microstructural changes during wire drawing of pearlitic steels. Mater. Sci. Eng. A.

[7] Wilson A.D. (1997). The influence of thickness and rolling ratio on the inclusion behaviour in plate steels. Metallography.

[8] Toribio J., Ayaso F.J. (2003). Anisotropic fracture behaviour of cold drawn steel: A materials science approach. Mater. Sci. Eng. A.

[9] Toribio J., González B., Matos J.C. (2010). Fatigue and fracture paths in cold drawn pearlitic steel. Eng. Fract. Mech..

[10] Rozumek D., Marciniak Z., Lesiuk G., Correia J.A., de Jesús A.M. (2018). Experimental and numerical investigation of mixed mode I+ II and I+ III fatigue crack growth in S355J0 steel. Eng. Fail. Anal..

[11] Macek W., Robak G., Żak K., Branco R. (2022). Fracture surface topography investigation and fatigue life assessment of notched austenitic steel specimens. Eng. Fail. Anal..

[12] Huang Y., Wang X. (2022). On the fracture toughness testing for single-edge notched bend specimen of orthotropic materials. Compos. Struct..

[13] Manjula S., Arun K.V. (2022). Environmentally assisted fracture behavior of edge and corner notched spring steel. Mater. Today Proc..

[14] Ayatollahi M.R., Torabi A.R. (2010). Tensile fracture in notched polycrystalline graphite specimens. Carbon.

[15] Gao S., Qi L., Zhu Y., Wang W. (2022). Effect of notch depth ratio on mode I and mixed mode I-II fracture properties of engineered cementitious composites (ECC). Int. J. Solids Struct..

[16] Çelik Z., Bingöl A.F. (2020). Fracture properties and impact resistance of self-compacting fiber reinforced concrete (SCFRC). Mater. Struct..

[17] Ruse N.D., Troczynski T., MacEntee M.I., Feduik D. (1996). Novel fracture toughness test using a notchless triangular prism (NTP) specimen. J. Biomed. Mater. Res..

[18] Shan D., Zhao M., Shao H., Fang C. (2019). Fracture behavior of notched TC21 alloy observed by In-situ SEM. Results Phys..

[19] Nazarova E.D., Filin V.Y., Galchun I.A. (2022). On the problem of getting a correct crack shape in fracture toughness specimens of low-carbon steel. Mater. Sci. Forum.

[20] Feng R., Chen M., Xie L. (2024). Constitutive relationship and fracture mechanism for wide stress triaxiality of titanium alloy. Eng. Fract. Mech..

[21] Torabi A.R., Ghanbari A., Choupani N., Ayatollahi M.R. (2024). Fracture assessment of blunt V-notched 3D-printed ABS: Proposing a new specimen for testing and different criteria for prediction. Theor. Appl. Fract. Mech..

[22] Toribio J., Ayaso F.J. (2002). Fracture process zone in notched samples of cold drawn pearlitic steels. ISIJ Int..

[23] Toribio J., Ayaso F.-J., Rodríguez R. (2023). Intercolonial microdamage and cracking micromechanisms during wire drawing of pearlitic steel. Materials.

[24] Kwon J.H., Heo J.M., Nguyen N.T., Tran M.T., Lee H.W., Kang S.H., Kim D.K. (2024). Ductile fracture locus under various deformation modes with negative-to-positive stress triaxiality. Int. J. Mech. Sci..

[25] Gao Y., Shao X., Wang Y., Hou P., Gao M. (2024). Does double pre-notches have a greater impact than single pre-notch on the mechanical and fracture behavior of rock? Insights from three-point bending tests using the numerical approach of grain-based model. Theor. Appl. Fract. Mech..

[26] He D., Chen X., Lin Y.C., Yan X., Xie H. (2024). Influences of stress triaxiality and forming parameters on microstructural evolution and fracture mechanisms in a Ni–Cr–Mo-based superalloy. J. Mater. Res. Technol..

[27] Komori K. (2025). Ductile fracture prediction during metal forming using an ellipsoidal void model and some other models. ISIJ Int..

[28] Toribio J., González B., Matos J.C. (2024). Notch tip hydrogen diffusion assisted by stress and strain fields and its role in hydrogen assisted fracture. Procedia Struct. Integr..

[29] Lin M., Yu H., Wang D., Díaz A., Alvaro A., Olden V., Zhang Z. (2024). Experimental and numerical study on hydrogen-induced failure of X65 pipeline steel. Mater. Sci. Eng. A.

[30] Liu L., Li L., He J., Liang Z., Peng Z., Gao J., Huang M. (2024). The unexpected low fracture toughness of dual-phase steels is caused by ferrite/martensite interface decohesion. Scr. Mater..

[31] Peng Z., Zhao H., Li X. (2021). New ductile fracture model for fracture prediction ranging from negative to high stress triaxiality. Int. J. Plast..

[32] Toribio J., Ayaso F.J. (2009). Micro-fracture maps in progressively drawn pearlitic steels under triaxial stress states. Int. J. Mater. Eng. Innov..

[33] Toribio J. (2020). On the concept of micro-fracture map (MFM) and its role in structural integrity evaluations in materials science and engineering: A Tribute to Jorge Manrique. Procedia Struct. Integr..

